# Mechanisms of Activation and Serotonin Release From Human Enterochromaffin Cells

**DOI:** 10.1016/j.jcmgh.2025.101610

**Published:** 2025-08-13

**Authors:** Constanza Alcaino, Nunzio Guccio, Emily L. Miedzybrodzka, Jaden R. Quale, Tianyi Lu, Adam Davison, Christopher A. Smith, Emily Overington, Marta Santos- Hernández, Mae Tabbada, Megan Hodge, Rula Bany Bakar, Richard Kay, Ahmed Shaaban, Cordelia Imig, Frank Reimann, Fiona M. Gribble

**Affiliations:** 1 Institute of Metabolic Science Metabolic Research Laboratories, Addenbrooke’s Hospital, Cambridge, United Kingdom; 2 Department of Neuroscience, University of Copenhagen, Copenhagen, Denmark

**Keywords:** Electrophysiology, Imaging, Organoid, Transcriptomics

## Abstract

**Background & Aims:**

Gastrointestinal (GI) enterochromaffin (EC) cells are specialised sensors of luminal stimuli. They secrete most of the body’s serotonin (5-HT), and are critical for modulating GI motility, secretion, and sensation, while also signaling satiety and intestinal discomfort. The aim of this study was to investigate mechanisms underlying the regulation of human EC cells, and the relative importance of direct nutrient stimulation compared with neuronal and paracrine regulation.

**Methods:**

Intestinal organoids from human duodenal biopsies were modified using CRISPR-Cas9 to specifically label EC cells with either the fluorescent protein Venus or the cyclic adenosine monophosphate (cAMP) sensor Epac1-S-H187. EC cells were purified by fluorescence-activated cell sorting for analysis by bulk RNA sequencing and liquid chromatography mass spectrometry peptidomics. The function of human EC cells was studied using single-cell patch clamp, calcium and cAMP imaging, and 5-hydroxytryptamine (5-HT) enzyme-linked immunosorbent assays (ELISAs).

**Results:**

Human EC cells showed expression of receptors for nutrients (including *GPR142*, *GPBAR1, GPR119, FFAR2, OR51E1, OR51E2*), gut hormones (including *SSTR1,2&5*, *NPY1R, GIPR*) and neurotransmitters (*ADRA2A*, *ADRB1*). Functional assays revealed EC responses (calcium, cAMP, and/or secretion) to a range of stimuli, including bacterial metabolites, aromatic amino acids, and adrenergic agonists. Electrophysiological recordings showed that isovalerate increased action potential firing.

**Conclusions:**

5-HT release from EC cells controls many physiological functions and is currently being targeted to treat disorders of the gut-brain axis. Studying ECs from human organoids enables improved understanding of the molecular mechanisms underlying EC cell activation, which is fundamental for the development of new strategies to target 5-HT-related gut and metabolic disorders.

## Graphical abstract

**Figure:**
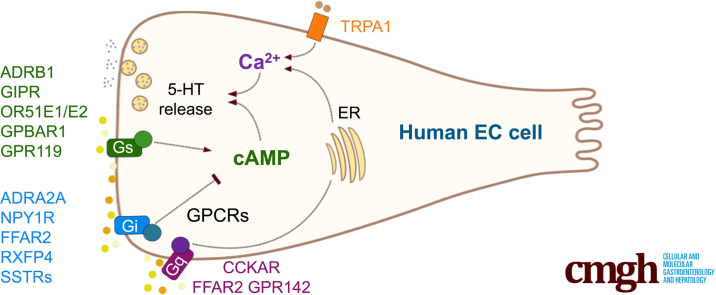

**Summary**

Human duodenal organoids expressing fluorescent proteins in enterochromaffin cells were used to study mechanisms underlying serotonin secretion. Different expression of key sensory receptors was identified by transcriptomic analysis and validated by live cell second messenger imaging and secretion assays.

Enterochromaffin (EC) cells are the largest population of enteroendocrine cells (EECs) in the intestine and produce most of the body’s systemic serotonin (5-hydroxytryptamine [5-HT]). This 5-HT is critical for normal gastrointestinal (GI) function, modulating intestinal secretion, motility, and sensation, while also conveying a signal that modulates food intake.^1^ Platelets take up 5-HT released from the gut and act as a high-capacity reservoir of 5-HT in the circulation, making it difficult to monitor acute release of 5-HT from EC cells in vivo, against this high background. Our understanding of the physiological stimuli controlling EC secretion, particularly in humans, is therefore relatively limited and mostly derived from studies on isolated cells and cell lines.

A number of studies have shown that EC cells are modulated by chemical and mechanical cues,^2^, ^3^, ^4^ but it is debated whether small intestinal 5-HT secreting EC cells are directly nutrient-sensitive. A study in dogs reported elevation of 5-HT in portal blood after instillation of hypertonic glucose into the small intestine, but did not conclude whether it was glucose itself or the hypertonicity and consequent gut distension that acted as the trigger of 5-HT release.^5^ Another study reported elevated circulating 5-HT levels in humans after ingestion of carbohydrates and fats, but suggested that the slow time course of the rise after the carbohydrate meal was more likely to reflect changes in peripheral 5-HT production or clearance than release from the gut.^6^ Free sugars and fatty acids were, however, shown to trigger 5-HT secretion from intestinal biopsies and purified EC cell populations.^7^, ^8^, ^9^, ^10^ mRNA expression levels of nutrient-sensitive receptors in EC cells have been reported to be location-dependent and to vary between species,^3^^,^^8^^,^^11^^,^^12^ although one study suggested that small intestinal EC cells lack nutrient-sensing machinery and rely on paracrine signaling from L-cell-derived glucagon-like peptide 1 (GLP-1).^11^

Murine EC cells have also been shown to be responsive to irritant-activators of Trpa1 channels, to short and branched chain fatty acids (SCFA, BCFA) and to adrenergic receptor stimulation.^3^ EC cell activation sensitizes sensory afferent neurons to mechanical stimulation and is critical for mechanically induced visceral hypersensitivity in mice.^13^ In humans, diets low in fermentable oligosaccharides, disaccharides, monosaccharides and polyols (FODMAPs) reduce pain and diarrhea in some patients with irritable bowel syndrome, potentially by reducing the action of microbially produced SCFA on EC cells.^14^ Germ-free mice have blunted expression of *Tph1* (tryptophan hydroxylase—a key step in 5-HT biosynthesis in EC cells) and colonic 5-HT content,^15^ suggesting that microbiota-derived metabolites such as SCFAs can also modulate EC cell numbers or expression profiles.^16^^,^^17^

The extent to which EC cell secretion is regulated by local paracrine signals remains unclear, although candidate local paracrine modulators have been suggested previously. Somatostatin (SST), which inhibits other EEC subtypes such as L-cells,^18^ also reduced 5-HT release from the carcinoid cell line BON, often used as an EC model cell line.^19^ In the colon, a few studies have shown that insulin-like peptide 5 (INSL5), a hormone co-secreted from L-cells, stimulates colonic motility through a pathway sensitive to 5-HT_3_ receptor inhibition, suggesting potential cross-talk with local EC cells; however, although the INSL5 receptor Rxfp4 is expressed by mouse EC cells, the preferential G_i_-coupling of this receptor would suggest inhibition rather than stimulation of 5-HT secretion from EC-cells.^20^^,^^21^

In this study, we aimed to characterize the mechanisms underlying 5-HT secretion from human EC cells. We developed transgenic EC-labeled organoids derived from human duodenal biopsies, using CRISPR-Cas9 homology-directed repair (HDR) to express fluorescent proteins specifically in cells expressing *TPH1*. Fluorescently labeled EC cells from these organoids were purified by flow cytometry for bulk RNA sequencing and peptidomic analysis, defining the expression repertoire of EC-cells. We used live cell fluorescent Ca^2+^ and cyclic adenosine monophosphate (cAMP) imaging, supported by 5-HT secretion measurements from intact organoids, to examine whether receptors identified by RNA sequencing elicited functional responses to stimuli such as nutrients, neurotransmitters, and hormones. The ion channel expression profile and previous reports from other EECs suggested that EC cells are electrically excitable; this was confirmed using patch clamp recordings, which were also used to monitor vesicular release as changes in cell capacitance. This work provides the first functional characterization of primary (non-tumor-derived) human EC cells, which is critical for our understanding of their role in disorders of the gut-brain axis.

## Results

### Development and Characterization of TPH1-Venus+ EC Cells From Human Duodenal Organoids

**Figure 1:**
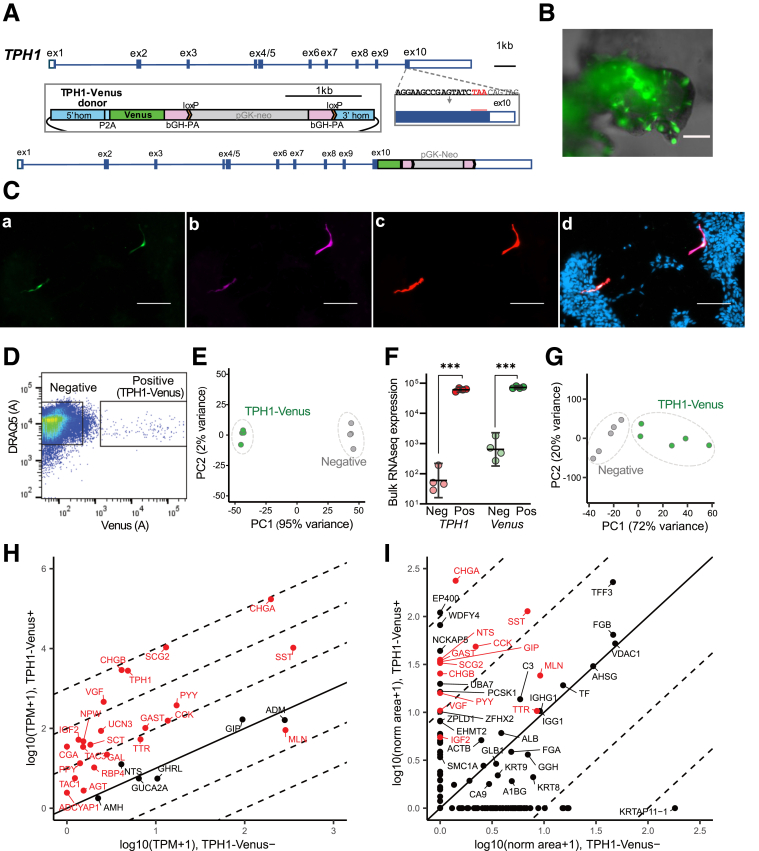
**Development and expression profile of human duodenal EC cells.** (*A*) Schematic showing the knock-in strategy to insert YFP Venus gene in exon 10 of the *TPH1* gene using CRISPR-Cas9 HDR. The same strategy was used to insert an Epac1-S-H187 sensor for cAMP measurements. (*B*) Image of duodenal 3D organoid from TPH1-Venus line. Venus+ cells are shown in *green*. Scale bar represents 400 μm. (*C*) Immunocytochemistry of fixed TPH1-Venus human duodenal 2D epithelial monolayer cultures. Cells staining positive for (*a*) GFP/Venus (*green*) are also immunopositive for (*b*) 5-HT (*magenta*) and (*c*) Chromogranin A (*red*). (*d*) Merged image of *a–c* with DAPI nuclear staining. Scale bars represent 100 μm. (*D*) Representative FACS plot. TPH1-Venus-positive and -negative cells were isolated based on Venus fluorescence intensity, after selection for live DAPI-negative and DRAQ5-positive cells only. (*E*) PCA plot showing TPH1-Venus-positive (*green*) and -negative (*gray*) cell populations following bulk RNAseq analysis. (*F*) Expression of *TPH1* and *Venus* genes in sorted Venus-positive and -negative populations following bulk RNA sequencing analysis. Lines indicate the mean ± 95% CI; comparisons between positive and negative cells were made by *t*-test; ∗∗∗*P* < .001. (*G*) PCA plot showing TPH1-Venus-positive (*green*) and -negative (*gray*) cell populations following peptidomic analysis. (*H*) Transcriptomic analysis plot showing expression of genes of interest in TPH1-Venus-positive (y-axis) vs TPH1-Venus-negative populations (x-axis). (*I*) LC-MS/MS peptidomic analysis of purified TPH1-Venus-positive and -negative cells (the individual peptides detected are combined and associated to the parental protein, labeled by protein name and expressed as mean peak area). Proteins of interest are highlighted in *red*.

To selectively label 5-HT-releasing human EC cells, we generated duodenal organoids from patient-derived biopsies and used CRISPR-Cas9-mediated HDR to express the yellow fluorescent protein Venus under the control of the *TPH1* promoter (Figure 1*A*). Resultant TPH1-Venus organoids exhibited sporadic green fluorescent cells, with a pattern and morphology consistent with fluorescent labeling of EC cells (Figure 1*B*). To validate the model and assess the morphology of human organoid-derived EC cells, 2-dimensional (2D) monolayer cultures from TPH1-Venus organoids were immunostained using antibodies against green fluorescent protein/Venus, 5-HT, and chromogranin A (Figure 1*C*), which confirmed specific expression of Venus in cells staining positive for 5-HT and chromogranin A.

Venus positive and negative cells were purified by fluorescence-activated cell sorting from organoid cultures, for bulk RNA sequencing and mass spectrometry peptidomics (Figure 1*D–I*). Principal component analysis (PCA) of the RNA-seq results showed clear separation of the fluorescent and nonfluorescent cell populations (Figure 1*E*). The expression of both *TPH1* and *Venus* were significantly higher in the *TPH1*-Venus+ fraction, compared with nonfluorescent cells, further validating that the fluorescent reporter marked EC cells (Figure 1*F*). In addition to *TPH1*, the fluorescently labeled cells were enriched for the expression of other gut hormones, such as the expected *TAC1*, a known EC cell peptide precursor, and also for peptide YY (*PYY)*, *SST,* and cholecystokinin (*CCK*) (Figure 1*H*). PCA of the liquid chromatography-tandem mass spectrometry (LC-MS/MS) peptidomic analysis showed a separation of the fluorescent and nonfluorescent cell populations (Figure 1*G*), and functional translation of gut hormone mRNAs was confirmed, as enriched production of chromogranin A, CCK, PYY, and SST was observed in the EC cell population (Figure 1*I*).

### Expression of Neurotransmitter Receptors in Human EC Cells

**Figure 2:**
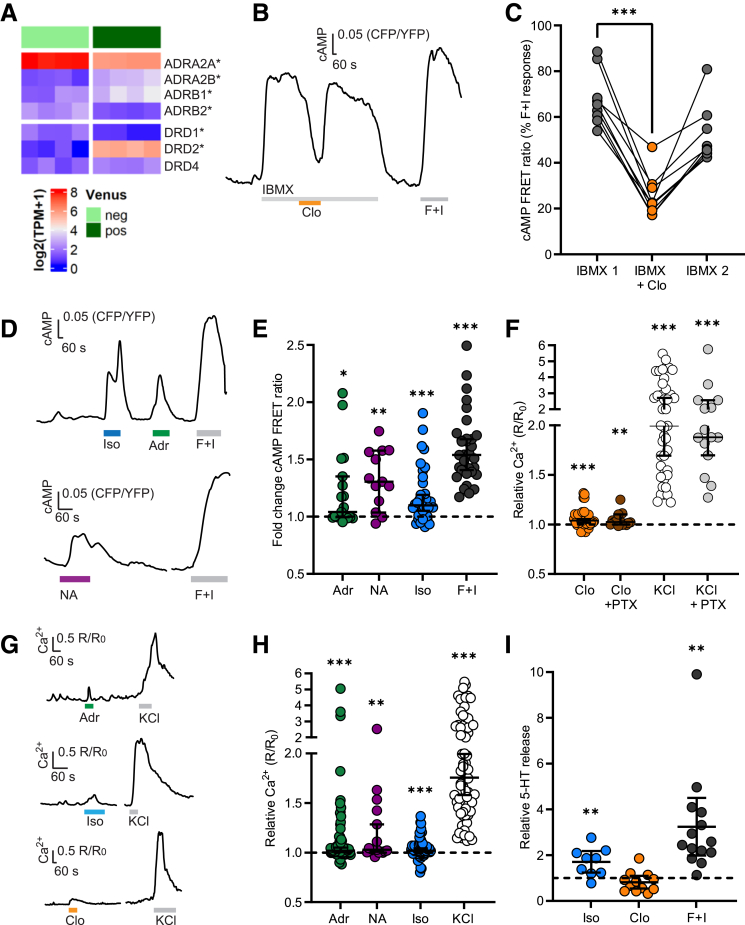
**Adrenergic activation of human EC cells.** (*A*) Heatmap showing expression of adrenergic and dopaminergic receptors in TPH1-Venus-positive (*dark green*) and -negative (*clear green*) cell populations. ∗Denotes significant enrichment in Venus+ population by Wald test (*P* < .05). (*B*) Representative FRET (CFP/YFP) ratio trace for single EC cell perfused with a preincubation of IBMX (100 μmol/L), followed by clonidine (Clo; 1 μmol/L) and positive control forskolin + IBMX (10 μmol/L and 100 μmol/L, respectively; F+I). (*C*) Data collected as in (*B*), shown as the maximum CFP/YFP ratios during perfusion of IBMX1 (before clonidine addition) and IBMX2 (after clonidine washout) and the minimum CFP/YPF ratio during perfusion of IBMX + clonidine (Clo), all expressed as a percentage of the response to F+I recorded in the same cell. ∗∗∗*P* < .001 by Friedman test with post hoc Dunn’s multiple comparisons test. (*D*) Representative FRET (CFP/YFP) ratio traces of single EC cells being perfused with (*E*) isoproterenol (Iso; 1 μmol/L), adrenaline (Adr; 30 μmol/L), noradrenaline (NA; 30 μmol/L), and Forskolin + IBMX (F+I; 10 μmol/L and 100 μmol/L, respectively). (*E*). Fold change of FRET ratio across different cells recorded as in (*D*). Data are shown as ratio between maximal CFP/YFP ratio (R) during perfusion of stimulus and maximal CFP/YFP ratio (R_0_) during perfusion of basal solution. (n = 13–34 cells from 2–4 dishes). Lines indicate the median ± 95% CI; ∗*P* < .05; ∗∗*P* < .01; ∗∗∗*P* < .001 by 1-sample Wilcoxon test. (*F*) Data showing the fold change in R/R_0_ (R being the Fura2 ratio during perfusion of the stimulus and R_0_ being the Fura2 ratio during perfusion of basal solution) in response to clonidine (Clo; 1 μmol/L) or KCl (70 mmol/L), following preincubation for 24 hours at 37°C with either control media or media treated with 200 ng/mL pertussis toxin (PTX). Lines indicate the median ± 95% CI; *P* = .39 for comparison between clonidine and clonidine + PTX by Mann-Whitney test. ∗∗*P* < .01 and ∗∗∗ *P* < .001 by 1-sample Wilcoxon test. (*G*) Representative Fura2 (340/380 nm) ratio traces showing responses of single EC cells perfused with adrenaline (Adr; 30 μmol/L), isoproterenol (Iso; 1 μmol/L), and clonidine (Clo; 1 μmol/L), with KCl (70 mmol/L) as a positive control. (*H*) Data from multiple cells recorded as in (*G*), or with noradrenaline (NA; 30 μmol/L), shown as ratio between R (Fura2 ratio during perfusion of stimulus) and R_0_ (Fura2 ratio during perfusion of basal solution). (n = 17–72 cells from 14–60 dishes). Lines indicate the median ± 95% CI; ∗∗*P* < .01; ∗∗∗*P* < .001 by 1-sample Wilcoxon test. (*I*) Fold change of supernatant 5-HT concentrations in human duodenal TPH1-Venus-positive 3D organoids incubated for 1 hour with isoproterenol (Iso; 1 μmol/L), clonidine (Clo; 1 μmol/L), and forskolin + IBMX (F+I; 10 μmol/L and 100 μmol/L, respectively). Results are presented relative to concentrations measured in control wells from the same plate. Lines indicate the mean ± 95% CI; ∗∗*P* < .01 by 1-sample *t*-test.

Human TPH1-Venus (EC) cells expressed a variety of neurotransmitter receptors, including GABA, dopamine, acetylcholine, and adrenergic receptors (Figure 2*A* and deposited RNA sequencing [RNAseq] data), suggesting modulation of EC cells by both intrinsic and extrinsic neurons found in the gut epithelium, consistent with previous reports.^3^^,^^22^ Contrasting with the previously reported expression profile of mouse EC cells identified as *ChgA*-positive cells, however, which emphasised a lack of beta-adrenergic receptor expression,^3^ human EC cells exhibited detectable and enriched expression of *ADRB1* in addition to *ADRA2A, ADRA2B,* of which *ADRA2B*, but not *ADRA2A* was enriched in fluorescent compared with nonfluorescent cells, even though *ADRA2A* displayed the highest expression (Figure 2*A*). Expression of ADRA1 receptors was below the limit of detection. Dopamine receptors, known to also be partially activated by adrenergic agonists, were also expressed in human EC cells, with *DRD2* notably enriched in the TPH1-fluorescent population.

To understand the contribution of the different expressed adrenergic receptors, which are preferentially Gs (ADRB-receptors) or Gi (ADRA2-receptors) coupled and thus should affect cytosolic cAMP levels, to the previously reported stimulation of 5-HT secretion by adrenaline, we developed a TPH1-Epac1-S-H187 organoid line to measure changes in intracellular cAMP concentrations. Epac1-S-H187 enables single live cell monitoring of cAMP changes through fluorescence resonance energy transfer (FRET) imaging in 2D organoid cultures, as previously reported in a GIP-Epac1-S-H187 duodenal organoid line.^23^ The selective ADRA2 agonist clonidine (1 μmol/l) reduced cAMP levels in EC cells in the presence of the nonselective phosphodiesterase inhibitor 3-isobutyl-1-methylxanthine (IBMX, 100 mmol/L) (Figure 2*B,C*), whereas application of the ADRB1/2 agonist isoproterenol (1 μmol/L) increased cAMP levels (median 1.10-fold; interquartile range [IQR], 1.00–1.31) FRET change; *P* < .001) (Figure 2*D, E*). The nonselective adrenoreceptor agonists adrenaline (30 mmol/L) and noradrenaline (30 mmol/L) also significantly elevated EC cell cAMP levels (FRET changes of 1.04-fold; IQR, 1.00–1.35; *P* = .036; 1.31-fold; IQR, 1.09–1.57; *P* = .002; respectively) (Figure 2*D, E*).

A previous study on murine EC cells demonstrated that activation of the Gi-coupled Adra2a could increase intracellular Ca^2+^ concentrations by opening the transient receptor potential channel Trpc4. We therefore tested the response to ADRA2 activation in human EC cells using the ratiometric Ca^2+^ dye Fura2-AM, revealing that even though clonidine (1 μmol/L) decreased cAMP, it induced a small but significant increase in the Fura2 ratio, indicative of an increase in the intracellular Ca^2+^ concentration (median, 1.04; IQR, 1.02–1.10; Fura2 R/R_0_; *P* < .001; 23/42 cells exhibited significant responses by z-score analysis). However, significant clonidine responses were also observed after blocking Gai-coupling with pertussis toxin (PTX) (1.03; IQR, 1.00–1.10; Fura2 R/R_0_; *P* = .004; 10/15 cells responded) (Figure 2*F*). EC cell Ca^2+^ responses were additionally observed in response to adrenaline (30 mmol/L; 1.02; IQR, 1.00–1.16; Fura2 R/R_0_; *P* < .001; 19/44 cells responded), noradrenaline (30 mmol/L; 1.03; IQR, 1.01–1.36; Fura2 R/R_0_; *P* = .001; 11/19 cells responded), and isoproterenol (1 μmol/L; 1.02; IQR, 0.99–1.07; Fura2 R/R_0_; *P* < .001; 14/38 cells responded) (Figure 2*G, H*).

To study the effects of adrenergic receptor agonists on 5-HT release from human EC cells, we performed secretion assays on 3D organoids and measured 5-HT release by enzyme-linked immunosorbent assay (ELISA) after 60-minute test incubations. Isoproterenol (1 μmol/L), but not clonidine (1 μmol/L), elicited a significant increase in 5-HT release compared with baseline (Figure 2*I*) (mean ± standard deviation [SD], 1.7 ± 0.6-fold; *P* = .009; and 0.8 ± 0.4-fold; *P* = .18; respectively). Overall, the combined expression and functional data suggest that human EC cell activation by adrenergic agonists relies heavily on ADRB1, contrasting with the report of a predominant role for Adra2 in murine EC cells.^3^^,^^13^

### Paracrine Activation of Human EC Cells by Gut Hormones

**Figure 3:**
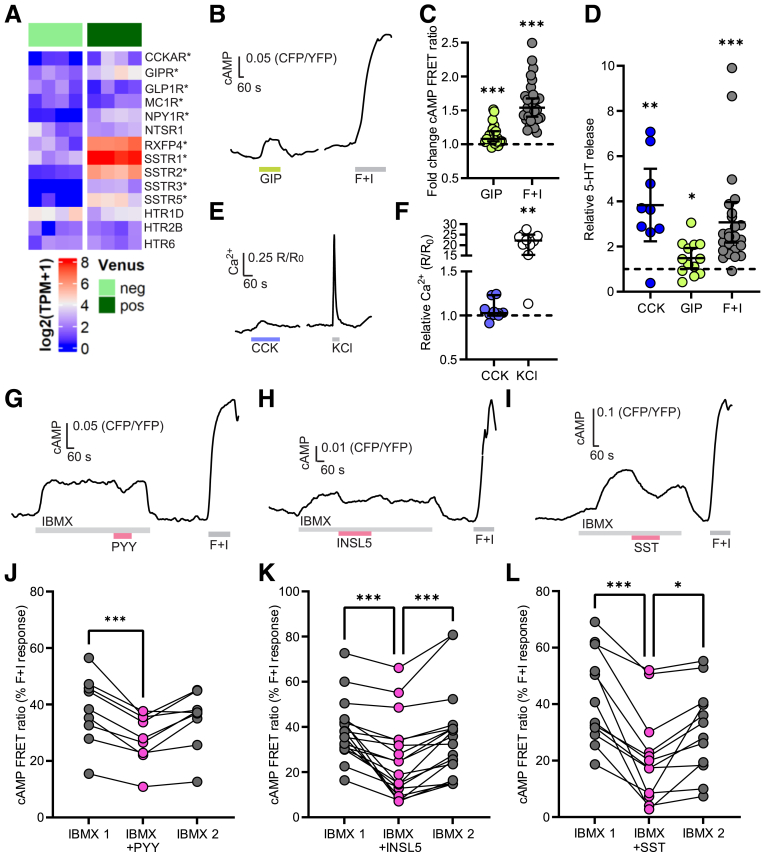
**Paracrine activation of human Enterochromaffin cells by gut hormones.** (*A*) Heatmap showing expression of selected peptide hormone and monoamine receptors in TPH1-Venus EC cells (*dark green*) compared with the Venus-negative population (*light green*) by bulk RNAseq. ∗Depicts significant enrichment in Venus+ population by Wald test (*P* < .05). (*B*) Representative FRET (CFP/YFP) ratio trace of single EC cell being perfused with GIP (100 nmol/L) followed by forskolin + IBMX (F+I; 10 μmol/L and 100 μmol/L, respectively). (*C*) Fold change of FRET ratios recorded as in (*B*) across n = 22 cells from 3 dishes). Lines indicate the median ± 95% CI; ∗∗∗*P* < .001 by 1-sample Wilcoxon test. (*D*) Fold change of supernatant 5-HT concentration in human duodenal TPH1-Venus-positive 3D organoids incubated for 1 hour with CCK (100 nmol/L), GIP (100 nmol/L), or forskolin + IBXM (F+I; 10 μmol/L and 100 μmol/L, respectively). Results are presented relative to concentrations measured in control wells from the same plate (n = 13–15 from 3 plates. Lines indicate the mean ± 95% CI; ∗P < .05; ∗∗∗*P* < .001 by 1-sample *t*-test. (*E*) Representative Fura2 (340/380 nm) ratio trace of a single EC cell being perfused with CCK (100 nmol/L) and KCl (70 mmol/L). (*F*) Increase in intracellular calcium levels across different cells recorded as in (*E*), shown as ratio between R (Fura2 ratio during perfusion of stimulus) and R_0_ (Fura2 ratio during perfusion of basal solution). (n = 10 from 9 dishes). Lines indicate the median ± 95% CI; ∗*P* < .05; ∗∗∗*P* < .001 by 1-sample Wilcoxon test. (*G–I*) Representative FRET traces of TPH1-Epac1-S-H187 EC cells perfused with IBMX (100 μmol/L), in the presence or absence of (*G*) PYY (100 nmol/L), (*H*) INSL5 (100 nmol/L), and (*I*) SST-14 (100 nmol/L). Forskolin (10 μmol/L) and IBMX (100 μmol/L) were used as positive control (F+I). (*J–L*) Responses of individual cells recorded as in (*G–I*). Ratios in IBMX in the presence or absence of PYY (*J*), INSL5 (*K*), and SST (*L*) are expressed as percentage of the forskolin + IBMX maximum response recorded in the same cell (n = 9–18 cells each, from 3–6 dishes). ∗*P* < .05; ∗∗∗*P* < .001 by Friedman test with post hoc Dunn’s multiple comparisons test.

We found differential expression of several gut hormone receptors in human EC cells, including the Gi-coupled SST receptors *SSTRs 1, 2, 3 and 5*, and the PYY receptor *NPY1R*, the Gq-coupled CCK receptor *CCKAR*, and the Gs-coupled incretin hormone receptor *GIPR* (Figure 3*A*). To our surprise, and despite INSL5 production being restricted to the distal colon and rectum, we found enriched expression of its receptor, *RXFP4,* in duodenal EC cells (Figure 3*A*). As GIPR is Gs-coupled, we tested the effect of 100 nmol/L glucose-dependent insulinotropic peptide (GIP) (1-42) on cAMP levels in EC cells, revealing significant cAMP responses (median FRET change, 1.08; IQR, 1.04–1.20; *P* < .001) (Figure 3*B, C*). GIP (100 nmol/L) also significantly stimulated 5-HT release (mean ± SD, 1.5 ± 0.7-fold above baseline; *P* = .035) (Figure 3*D*). CCK (100 nmol/L) was tested on cytoplasmic Ca^2+^ levels due to the known Gq coupling of CCKAR, revealing a small but significant Ca^2+^ response in 6 of 10 cells (1.02; IQR, 1.00–1.07; Fura2 R/R_0_; *P* = .042) (Figure 3*D, E*). CCK also stimulated a relatively robust 5-HT secretory response (3.8 ± 2.1-fold above baseline; *P* = .004) (Figure 3*D*). Activation of Gi-coupled receptors, assessed by cAMP FRET imaging, showed lowering of EC cell cAMP levels by SST, PYY, and INSL5 (Figure 3*G–L*).

### Human EC Cells Are Nutrient Sensors

**Figure 4:**
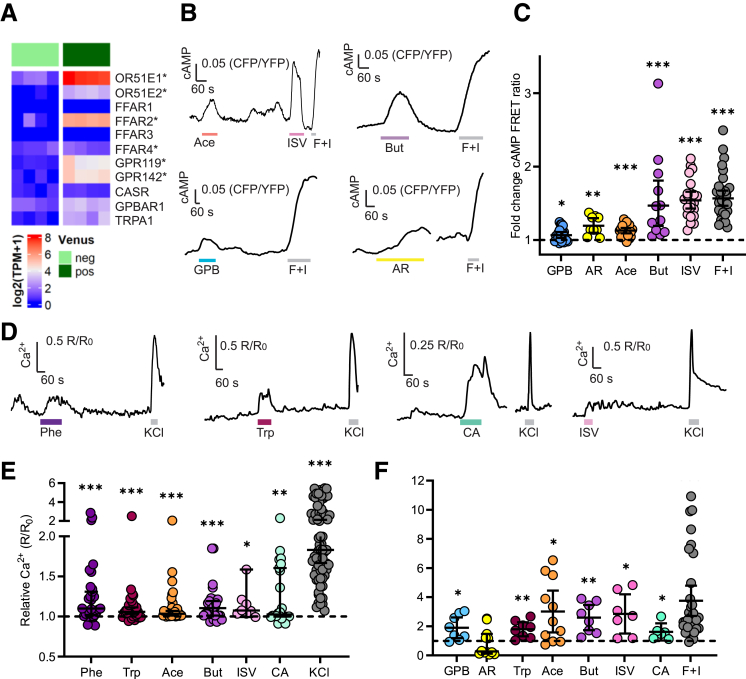
**Human EC cells are nutrient sensors.** (*A*) Heatmap showing expression of selected nutrient-sensing receptors in TPH1-Venus EC cells (*dark green*) compared with the Venus-negative population (*light green*) by bulk RNAseq. ∗Denotes significant enrichment in Venus+ population by Wald test (*P* < .05). (*B*) Representative FRET (CFP/YFP) ratio traces of single EC cells being perfused with acetate (Ace; 200 μmol/L), isovalerate (ISV; 200 μmol/L), butyrate (But; 2 mmol/L), GPBAR-A (GPB; 3 μmol/L), AR231453 (AR; 100 nmol/L) and forskolin + IBMX (F+I; 10 μmol/L and 100 μmol/L, respectively). (*C*) Fold change of FRET ratio across different cells recorded as in (*B*). (n = 9–22 cells from 1–3 dishes. Lines indicate the median ± 95% CI; ∗*P* < .05; ∗∗*P* < .01; ∗∗∗*P* < .001 by 1-sample Wilcoxon test. (*D*) Representative Fura2 (340/380 nm) ratio traces of single EC cells being perfused with phenylalanine (Phe; 20 mmol/L), tryptophan (Trp; 20 mmol/L), cinnamaldehyde (CA; 300 μmol/L), isovalerate (ISV; 200 μmol/L), and KCl (70 mmol/L). (*E*) Data from individual cells recorded as in (*D*) and shown as ratio between R (Fura2 ratio during perfusion of stimulus) and R_0_ (Fura2 ratio during perfusion of basal solution). (n = 8–62 cells from 3–8 dishes). Lines indicate the median ± 95% CI; ∗*P* < .05; ∗∗*P* < .01; ∗∗∗*P* < .001 by 1-sample Wilcoxon test. (*F*) Fold change of supernatant 5-HT concentration in human duodenal TPH1-Venus-positive 3D organoids incubated for 1 hour in the presence of GPBAR-A (GPB; 3 μmol/L), isovalerate (ISV; 200 μmol/L), acetate (Ace; 200 μmol/L), butyrate (But; 1 mmol/L), tryptophan (Trp; 20 mmol/L), cinnamaldehyde (CA; 300 μmol/L) and forskolin + IBXM (F+I; 10 μmol/L and 100 μmol/L, respectively). Results are presented relative to concentrations measured in control wells from the same plate. Lines indicate the mean ± 95% CI; ∗*P* < .05; ∗∗*P* < .01; ∗∗∗*P* < .001 by 1-sample *t*-test (n = 6–20 from 3 plates).

We identified differential expression in human duodenal EC cells of several G-protein coupled receptors (GPCRs) that could respond to nutrients and luminal factors after feeding (Figure 4*A*), including the olfactory receptors *OR51E1* and *OR51E2*, the SCFA receptor *FFAR2*, the 2-acylglycerol receptor *GPR119*, the aromatic amino-acid receptor *GPR142*, and the bile acid receptor *GPBAR1*. The calcium sensor *CASR*, which plays a role in amino acid sensing in GIP-expressing cells,^23^ and the long-chain fatty acid receptor *FFAR1* were only detected at very low levels in EC cells, whereas *FFAR4* was detected at higher levels but not differentially expressed.

Functionally, the bile acid receptor agonist GPBAR-A (3 mmol/L), the GPR119 agonist AR231453 (100 nmol/L), and the OR51E1 agonist isovalerate (200 mmol/L)^24^ significantly increased intracellular cAMP in EC cells (FRET changes, 1.04; IQR, 0.99–1.15; *P* = .01; 1.14; IQR, 1.09–1.32; *P* = .004; and 1.53; IQR, 1.34–1.67; *P* < .001, respectively), consistent with the known Gs coupling of these receptors (Figure 4*B, C*). Acetate is an agonist of both FFAR2 (Gi/q-coupled) and OR51E2 (Gs-coupled),^25^ and was therefore assessed for effects on both cAMP and Ca^2+^; our results showed that acetate (200 mmol/L) induced an increase in both cAMP (FRET change, 1.13; IQR, 1.06–1.20; *P* < .001) (Figure 4*B, C*) and Ca^2+^ (1.04; IQR, 1.01–1.11; Fura2 R/R_0_; *P* < .001; 22/28 cells responded) in human EC cells (Figure 4*D, E*). Butyrate (1 mmol/L), an agonist for FFAR2 and OR51E1, elicited large elevations in Ca^2+^ (1.11; IQR, 1.01–1.21; Fura2 R/R_0_; *P* < .001; 7/23 cells responded) and cAMP (FRET change, 1.36; IQR, 1.12–1.85; *P* < .001) (Figure 4*B–E*). GPBAR-A, acetate, butyrate, and isovalerate all induced significant increases in 5-HT release compared with baseline (1.9 ± 0.8; *P* = .02; 3.0 ± 2.1; *P* = .01; 2.6 ± 1.0; *P* = .003; and 2.8 ± 1.5-fold; *P* = .015, respectively) (Figure 4*F*). AR231453 did not induce a significant increase in 5-HT release compared with baseline (0.83 ± 0.92-fold).

Functional responses of the amino acid receptors CASR and GPR142 were tested on intracellular Ca^2+^ levels, due to the known Gq coupling of both receptors. The aromatic amino acids tryptophan (20 mmol/L) and phenylalanine (20 mmol/L) both triggered elevation of intracellular Ca^2+^ in EC cells (Fura2 R/R_0_: tryptophan, 1.06; IQR, 1.00–1.15; *P* < .001, with 17/28 cells responding; phenylalanine, 1.10; IQR, 1.00–1.36; *P* < .001, with 14/29 cells responding) (Figure 4*D, E*). Tryptophan induced a significant increase in 5-HT release (1.8 ± 0.6-fold; *P* = .006) (Figure 4*F*). These responses are likely mediated by GPR142, as the calcium sensing receptor CASR was expressed only at very low levels in EC cells, but we cannot exclude other yet to be identified receptors in aromatic amino acid sensing.

Finally, the irritant receptor *TRPA1*, previously identified to be expressed in mouse EC cells,^2^^,^^3^^,^^26^ was also enriched in human EC cells (Figure 4*A*). As TRPA1 is an ion channel linked to elevation of Ca^2+^, we tested the effect of the TRPA1 agonist cinnamaldehyde (CA, 300 mM) on intracellular Ca^2+^. Consistent with the *TRPA1* expression data, CA induced an increase in intracellular Ca^2+^ (1.03; IQR, 1.00–1.62; Fura2 R/R_0_; *P* = .007; 10/23 cells responded) and 5-HT release (1.6 ± 0.6-fold; *P* = .04) (Figure 4*D–F*).

### Nutrient-induced Excitability Changes in Human EC Cells

**Figure 5:**
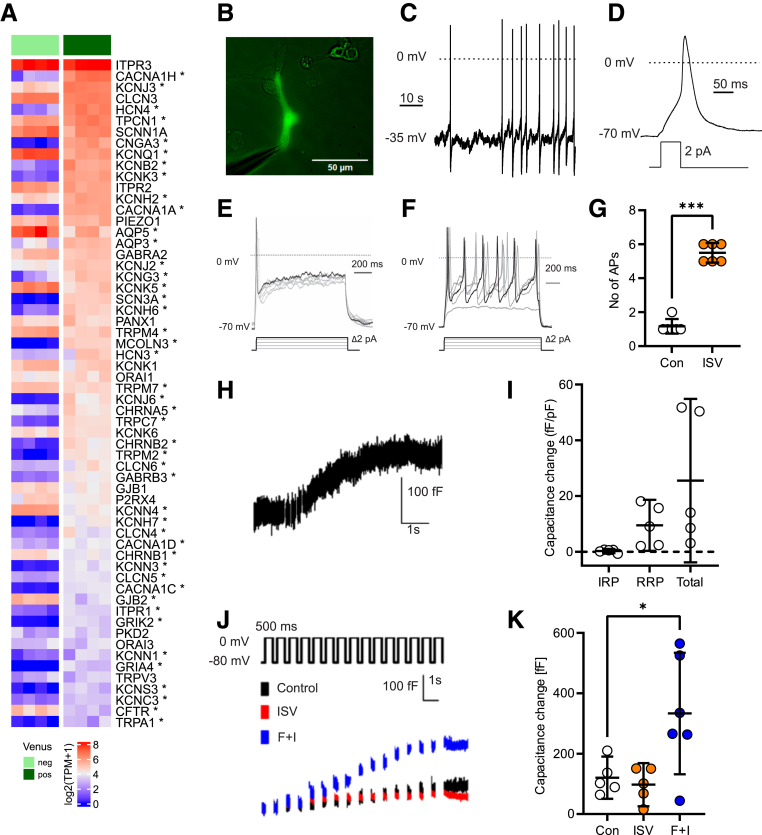
**Electrophysiological properties of human EC cells**. (*A*) Heatmap showing transcriptomic expression of top 60 ion channels from TPH1+ (*dark green*) and -negative (*clear green*) populations by bulk RNAseq, sorted by decreasing expression in the TPH1+ population. ∗Represents genes differentially expressed in Venus+ population by Wald test (*P* < .05). (*B*) Example image of human TPH1-Venus+ cell patch clamp experiment. (*C*) Example trace of spontaneous electrical activity of human TPH1-Venus+ cell recorded in perforated patch current clamp mode. (*D*) Example trace of action potential from human TPH1-Venus+ cell induced by current injection. Prior to the test pulse, the cell was held at −70 mV by current injection. (*E, F*) Example traces of current-induced action potentials (1-second pulse) from a single human TPH1-Venus+ cell recorded in control buffer (*E*) and in the presence of 200 μmol/L isovalerate (*F*). Cells were held at −70 mV by current injection prior to delivery of the pulse protocol indicated. (*G*) Individual data for cells recorded as in (*E, F*) showing number of action potentials (AP) triggered by the 30 pA injection pulse, in control buffer, and in the presence of 200 μmol/L isovalerate (ISV) during the 1-second current injection. Lines indicate the mean ± 95% CI; ∗∗∗*P* < .001 by paired *t*-test. (*H*) Example trace of provoked vesicular release (exocytosis) from a human TPH1-Venus+ cell. (*I*) Averaged provoked vesicular release (exocytosis) from human TPH1-Venus+ cells, showing normalized IRP, RRP, and total release, calculated as indicated in the methods. Lines indicate the mean ± 95% CI. (*J*) Representative traces of voltage-induced capacitance changes in control solution (Con), 200 μmol/L isovalerate (ISV), and forskolin + IBMX (F+I; 10 μmol/L and 100 μmol/L, respectively). (*K*) Combined data of voltage-induced capacitance changes from cells recorded as in (*J*). Lines indicate the mean ± 95% CI; ∗*P* < .05 by 1-way ANOVA with post hoc Dunnett’s multiple comparisons test.

Electrical excitability in different types of EEC, including EC cells, has been extensively studied,^3^^,^^17^^,^^27^, ^28^, ^29^ although there are no previous reports of the electrophysiological characteristics of human EC cells. Human EC cells differentially expressed the voltage-gated Na^+^ channel *SCN3A* (Nav1.3), voltage-gated Ca^2+^ channels *CACNA1H* (T-type/Ca_v_3.2), *CACNA1A* (P/Q-type/Ca_v_2.1), and *CACNA1C* (L-type/Ca_v_1.2), as well as the TRP channels *TRPC7* and *TRPM2* (Figure 5*A*). The mechanosensitive ion channel *PIEZO1* was expressed at similar levels in both EC cells and nonfluorescent control cells, whereas *PIEZO2* was not detected in either. We also found enrichment in EC cells of the hyperpolarization-activated nonselective cation channels *HCN4* and *HCN3*, the cyclic nucleotide gated channel *CNGA3*, and *KCNJ3* and *KCNJ6* (Kir3.1/Kir3.2) which together form a G-protein activated inwardly rectifying potassium channel (Figure 5*A*).

We recorded the electrical activity of single human EC cells from 2D organoid cultures (Figure 5*B*) using perforated patch clamp recordings and found that 9 of 9 cells fired spontaneous action potentials (resting membrane potential average, −7.9 ± 5.6 mV; n = 9) (Figure 5*C*). Properties of evoked action potential were similar to those previously reported in human K-cells^23^ and L-cells^30^ (action potential threshold, −23.5 ± 9.5 mV; overshoot, 35.5 ± 9.3 mV; and half-width, 19.1 ± 5.1 ms; n = 9) (Figure 5*D*). Acute treatment with isovalerate induced an increase in action potential firing elicited by 1-second long depolarizing current injections (1.2 ± 0.4 vs 5.5 ± 0.5 events per 1-second pulse; n = 6) (Figure 5*E−G*).

Whole cell voltage-clamp electrophysiological capacitance recordings were used to assess depolarization-induced exocytosis by human EC cells. To dissect distinct functionally defined secretory vesicle pools in human EC cells, we employed a depolarization protocol well-characterized for the study of exocytosis in adrenal chromaffin cells.^31^ Human EC cells had an average initial capacitance (a measure of cell size) of 10.8 ± 2.1 pF (n = 5). An example capacitance trace is shown in Figure 5*H*, and mean data from the 5 capacitance traces recorded using this protocol are shown in Figure 5*I*. We measured the “immediately releasable pool” (IRP) in response to a series of 6 short 10-ms depolarizations (3.5 ± 5.9 fF per cell [0.28 ± 0.61 fF/pF]), the “readily releasable pool” (RRP) after 4 additional long 100-ms depolarizations (91 ± 61 fF per cell [9.5 ± 7.4 fF/pF]), and the mean total vesicular release, determined as the change in membrane capacitance from 0 to 7 seconds (240 ± 199 fF per cell [25.6 ± 23.6 fF/pF]) (Figure 5*I*). Our findings indicate that human EC cells have a large functional secretory vesicle pool to support sustained exocytosis, similar to what has been reported for other endocrine cells, such as adrenal chromaffin cells.^31^

Next, we investigated if chemical stimuli could enhance exocytosis triggered by membrane depolarization. For this, we measured EC cell capacitance in response to a train of 500-ms depolarizing pulses from −80 mV to 0 mV in either regular extracellular solution or in the presence of 200 mmol/L isovalerate or forskolin + IBMX (10 mmol/L and 100 mmol/L, respectively) (Figure 5*J, K*). Pulse-evoked exocytosis was 121 ± 57 fF/cell (11.4 ± 6.2 fF/pF; n = 5) under control conditions and significantly elevated in the presence of forskolin + IBMX (333 ± 192 fF/cell; 21.8±10.5 fF/pF; n = 6). Isovalerate did not elevate exocytosis above control (97 ± 57 fF/cell; 9.2 ± 4.9 fF/pF; n = 5).

## Discussion

Human duodenal organoids expressing fluorescent proteins and sensors in EC cells provide a method to characterize the molecular mechanisms underlying gut 5-HT release—a question that has previously been hampered by a shortage of representative in vitro models and difficulties in measuring acute 5-HT release in plasma due to the high background storage of 5-HT in platelets. Like mouse EC cells and other human EECs, human EC cells were electrically active and exhibited enhanced exocytosis, measured by capacitance recordings, upon membrane depolarization and elevation of cAMP. Transcriptomic analysis of purified organoid-derived human duodenal EC cells identified expression of a range of GPCRs, which were functionally validated by fluorescence-based recordings of cytoplasmic Ca^2+^ and cAMP levels. The responsiveness of individual cells to specific stimuli was quite varied, which might reflect variation in receptor expression; however, we confirmed a population response by ELISA-based measurements of 5-HT secretion.

Although *TPH1* was the most highly enriched hormonal marker in human EC cells, the cells also exhibited enriched mRNA expression and detectable peptide levels of other gut hormones including SST, CCK, and PYY, although not of motilin (MLN) or GIP. This is consistent with previous reports that EC cells show transcriptomic overlap with other EEC populations.^32^^,^^33^ However, our recent single-cell RNAseq (scRNAseq) analysis of chromogranin-A-positive cells purified from human duodenal CHGA-Venus organoids, returned distinct EEC clusters showing only limited overlap between EC cells and cell populations clustering according to their expression of *SST*, *CCK,* and *PYY*, despite identifying coexpression of different hormones in a substantial proportion of cells.^34^ Consistent with studies on murine EC cells, we found enriched expression of tachykinin genes in human EC cells; however, unlike murine cells which predominantly express *Tac1*, we found both *TAC3* and *TAC1* in human cells, with *TAC3* expression being higher than that of *TAC1*. *TAC1* encodes Neurokinin A (NKA) and Substance P, whereas *TAC3* encodes Neurokinin B (NKB). Interestingly, a recent study reported that polymorphisms in *NK2R* (the cognate receptor for NKA > NKB) are associated with body weight and glucose control in humans, and that agonism of NK2R in mouse models resulted in reduced food intake, lower body weight, and loose stools.^35^ The physiological role of EC-derived tachykinin peptides in humans is not clear, although substance P is implicated in pain perception in the intestine.^36^

Among the GPCRs identified and characterized in human EC cells were members of the adrenergic receptor family, responsive to adrenaline and noradrenaline. Adrenergic stimulation of 5-HT release from human organoid-derived EC cells was predominantly mediated by β-adrenergic receptors, as also previously reported from measurements of portal blood 5-HT in cats after stimulation of sympathetic fibers carried in the vagus nerve.^37^ Human EC cells exhibited small calcium responses to α2 adrenoceptor agonism, mirroring findings in mouse EC cells. However, in mice, these have been attributed to Trpc4 channel activation downstream of Gi-activation, whereas in the human EC cells, they were insensitive to PTX.^3^ In human EC cells, Ca^2+^ responses to clonidine were accompanied by a reduction in cAMP levels, consistent with Gi activation, and no net stimulation of 5-HT secretion was observed. β-Adrenergic agonism with isoproterenol, by contrast, increased human EC cell Ca^2+^ and cAMP levels and 5-HT secretion. This appears to differ from murine EC cells, which have been reported not to express detectable β-adrenergic receptor mRNA transcripts or to exhibit responses to β-adrenergic agonism,^3^ although our recent scRNAseq analysis of murine EEC cells from different intestinal regions did detect b-adrenergic receptor expression in a number of EEC-clusters, including EC cells.^38^

Human EC cells exhibited detectable expression receptors for several gut hormones, including the Gs coupled receptors *GIPR* and *GLP1R*, the Gq coupled receptors *CCKR1* and *NTSR1*, and the Gi coupled receptors *NPY1R*, *RXFP4*, *SSTR1,2,3,* and 5, suggesting paracrine modulation of EC cells by other neighboring EECs. A number of these GPCRs were validated functionally; agonism of GIPR increased cAMP and enhanced 5-HT release, whereas PYY, SST, and INSL5 lowered cAMP in EC cells from human organoid cultures. Previous studies in proximal and distal mouse intestine also found evidence of paracrine communication between L-cells and EC cells,^11^^,^^39^ but as PYY, INSL5, and GLP-1 are coreleased by L-cells in response to nutrients,^40^, ^41^, ^42^, ^43^ it is surprising that these hormones have opposing effects on EC cells, with GLP-1 being stimulatory and PYY and INSL5 being inhibitory. Stimulation of mouse EC cells by GLP-1 was reported previously,^11^ and in vitro studies using a human epithelial cell line also reported that INSL5 decreased cAMP levels and inhibited 5-HT release.^21^ The in vivo balance of stimulatory and inhibitory interactions between L-cells and EC cells thus remains unclear, and direct effects of INSL5 on EC cells do not appear to account for observations that INSL5 administration or stimulation of INSL5-expressing L-cells induces a 5-HT-dependent increase in colonic motility in mice, as this would require stimulation not inhibition of EC cells by INSL5.^39^^,^^44^ It is possible that promotility effects of INSL5 in vivo require 5-HT-dependent signaling in the enteric nervous system, rather than release from EC cells.

Although previous studies suggested that EC cells do not express high levels of nutrient-sensing GPCRs, we found expression and functional activity of GPBAR1, GPR119, and GPR142 in human EC cells, potentially enabling the cells to respond to stimuli such as bile acids, monoacylglycerols, and aromatic amino acids.^23^ Another recent study of human organoid-derived EECs also investigated associations between GPCR expression and 5-HT (and other hormones) release using a receptor knockout approach.^45^ The authors reported effects of receptor agonists or antagonists on 5-HT secretion during 16-hour incubations and compared 5-HT in the supernatant in wild-type (WT) and knockout organoids to a control stimulation with forskolin and IBMX. Although several GPCR agonists stimulated a degree of 5-HT release, knockouts of *FFAR2, CASR, GPR142, GPR119,* or *GLP1R* did not significantly affect responses to their respective agonists. Exceptions were FFAR4, GPR19, and MC1R, which we have not further explored in our study, but our functional data presented here would support roles for FFAR2, GPR142, GPR119, and GIPR, whereas CASR seems not to be expressed to sufficient levels in EC cells. It is important to highlight that long-term exposure to these agonists in secretion experiments might additionally alter gene expression or activate paracrine signaling from neighboring EECs that will ultimately affect 5-HT secretion. Our functional assays additionally confirm that receptor agonists can directly activate single human EC cells via intracellular Ca^2+^ and/or cAMP.

When we compared human EC cells with other EEC populations using data from CHGA-Venus organoids,^34^ we found that expression levels of *GPBAR1*, *GPR119,* and *GPR142* in EC cells were similar to those in duodenal I-cells (CCK) and K-cells (GIP), whereas expression of *CASR* and *FFAR1* were notably lower in EC cells than in other EECs from the same intestinal region. Low expression of *FFAR1* is unusual for EECs^23^^,^^29^ and suggests a specific lack of FFAR1-dependent sensing of long chain fatty acids by EC cells. Orally ingested lipids could still, however, potentially stimulate 5-HT release via monoacylglycerols acting on GPR119, even though we failed to observed stimulated secretion to a GPR119 agonist in vitro, or by GPBAR1-dependent action of bile acids present in lipid micelles.

EC cells exhibited high expression of *OR51E1* and *FFAR2*, which are responsive to SCFAs and BCFAs produced by microbial fermentation. The olfactory receptors OR51E1 and OR51E2, and their mouse homologues Olfr558 and Olfr78, have been identified previously in mouse and human EECs, particularly in the lower GI tract where microbiota levels are high. The observed Ca^2+^ responses to acetate and butyrate in human EC cells are likely attributable to the Gi/q coupled receptor FFAR2, and the cAMP responses to butyrate and isovalerate likely reflect the Gs coupling of OR51E1. As OR51E1 is not considered sensitive to acetate, the observed acetate-triggered cAMP response may reflect OR51E2 activity. We were surprised, and cannot explain, that although isovalerate increased cAMP levels and 5-HT secretion, it did not enhance EC cell secretion measured by capacitance recordings; one possible explanation is that cAMP responses to forskolin + IBMX are larger and more sustained than those to isovalerate in the vicinity of secretory vesicles.

Alternatively, prolonged exposure to isovalerate might result in desensitization of the signals enhancing capacitance responses to strong depolarizing pulses, consistent with a detectable decline in the cAMP response during the short exposure shown in Figure 4*B*; however, the increased action potential frequency in response to depolarizing pulses was observed after exposure to isovalerate for several minutes, which might contribute to the increase in serotonin secretion during a 1-hour exposure.

## Conclusions

Human EC cells are modulated by a range of intestinal chemical stimuli, including sympathetic tone, paracrine actions of other gut hormones, ingested nutrients, and microbial metabolites—in addition to their previously-demonstrated responsiveness to mechanical forces. As EC cell activity contributes to the regulation of intestinal motility, conveys signals related to intestinal pain, and initiates pathways that regulate appetite, understanding the mechanisms controlling human EC cells provides opportunities to intervene therapeutically in a range of clinical disorders of the gut-brain axis.

## Methods

### Human Organoid Culture and Maintenance

Human duodenal organoids were derived from anonymous surgical samples from Addenbrooke’s Hospital Tissue Bank, under ethical approval by East of England–Cambridge Central Research Ethics Committee (license number 09/H0308/24). Duodenal organoids were generated and maintained as described previously.^23^ Cultures were maintained as domes of several 3D spheroids embedded in basement membrane extract (BME) on the base of tissue culture plates. Cultures were passaged every 7 days by adding 1 to 4 mature domes to TrypLE (ThermoFisher) at 37°C for 7 minutes and triturating via pipetting through a P1000 pipette 20 to 30 times. Residual small cell clusters were then embedded in BME and seeded onto prewarmed plates. To promote differentiation, EGF was removed from the culture medium 2 to 3 days after seeding (IF medium). Subsequently, organoids were grown in IF medium for 5 to 7 days, at which point fluorescent TPH1-Venus cells could be observed. For access to the Venus-positive cells for imaging and electrophysiological experiments, 2D monolayer cultures were made. For this, organoids were dissociated as described and seeded onto 2% Matrigel (Corning)-coated 35-mm plastic or glass coverslips and glass bottom dishes for electrophysiology and imaging experiments, respectively, and incubated overnight.

### Generation of TPH1-Venus Human Duodenal Organoids

CRISPR-Cas9 HDR was used to knock in either Venus or the FRET cAMP reporter Epac1-S-H187,^46^ following a P2A sequence to enable bicistronic expression under control of the TPH1 promoter (Figure 1*A*). A CRISPR site (ACTGGCTACTGTTAGATACTCGG) close to the stop codon (underlined) in exon 10 was targeted. SgRNA-Cas9 and donor plasmids were generated, purified, and prepared for electroporation, as described previously.^28^^,^^29^ Successful recombinants were enriched by antibiotic selection by adding G418 (0.5 mg/mL) to the medium 3 to 7 days after electroporation. Surviving organoids were manually picked and seeded in single BME domes to ensure establishment of monoclonal organoid lines. DNA was extracted from each organoid using QuickExtract DNA Extraction Solution (Lucigen Corporation), and successful integration was assessed by polymerase chain reaction (PCR) genotyping and confirmed by Sanger sequencing (Source Bioscience).

### cDNA Library Preparation and RNAseq

RNA extraction and sequencing were performed after fluorescence-activated cell sorting (FACS) as previously described.^28^^,^^29^ Venus-fluoresecent (TPH1+) and nonfluorescent (negative) cells were collected from 4 independent sorts: FACS1: 2885 TPH1+ and 3000 negative cells; FACS2: 7139 TPH1+ and 7000 negative cells; FACS3: 13,812 TPH1+ and 13,500 negative cells and FACS4: 4423 TPH1+ and 5000 negative cells. Only genes with mean >10 reads across all samples were included in downstream analysis. In the final dataset, there were 9.69 to 22.0 million reads per library. Principle component analysis (PCA) was performed using DESeq2’s plotPCA function, after variance-stabilizing transformation. Differential expression compared TPH1-Venus positive vs negative cell populations and was calculated using the Wald test and subsequent Benjamini-Hochberg multiple testing correction (default in DESeq2), where significance was defined at *P*_adj < .05. Gene expression is presented in transcripts per million (TPM). Raw and processed data files are available in the Gene Expression Onmibus (GEO) database (GSE292419).

### Peptidomic Analysis of Sorted EC Cells

Peptide extraction and analysis of FACS-sorted cells were performed by LC-MS/MS as previously described.^28^^,^^29^ 10,000 Venus-fluoresecent (“TPH+”) and 10,000 nonfluorescent (negative) cells were collected from each of 5 independent sorts. Sorted cells collected in 250 μL 6 M guanidine hydrochloride were snap frozen on dry ice then subjected to 3 freeze/thaw events before 900 μL of 80% acetonitrile in water was added and mixed thoroughly by vortexing. The aqueous layer (lower) was removed and evaporated in a rotary evaporator (Eppendorf) for 18 hours. The residue was reconstituted with 500 μL of 0.1% formic acid (FA) in water (v/v) then extracted by solid phase extraction (SPE), evaporated, reduced, and alkylated, as previously described, prior to LC-MS analysis. Prior to injection onto the LC system, samples were diluted with 25 μL 1% FA in water (v/v) and centrifuged (3500 g, 10 minutes). Peptide extracts were analysed using a Thermo Fisher Ultimate 3000 Nano LC system coupled to a Q Exactive Plus Orbitrap mass spectrometer (Thermo Scientific). Samples (40 μL) were loaded onto a 0.3 × 5 mm peptide trap column at a flow rate of 30 μL/min and washed for 15 minutes before switching in line with a 0.075 × 250 mm nano easy column flowing at 300 nL/min. Both nano and trap column temperatures were set at 45°C. The mobile phases were A: 0.1% FA in water (v/v) and B: 0.1% FA (v/v) in 80:20 ACN:water. Initial conditions were 2.5% B and held for 15 minutes. A ramp to 50% B was performed over 90 minutes, and the column then washed with 90% B for 20 minutes before returning to starting conditions for a further 20 minutes, totaling an entire run time of 130 minutes. Positive nano electrospray analysis was performed using a spray voltage of 1.8 kV, and an S-lens setting of 70 V. A full scan range of 400 to 1600 m/z was performed at a resolution of 75,000 before the top 10 ions of each spectrum were selected for MS/MS analysis. Existing ions selected for fragmentation were added to an exclusion list for 30 seconds.

Peptidomic searches of the Human Uniprot database were performed using PEAKS (v8.5, BSI) and the peak integration was performed using Xcalibur (v4.3.73.11, Thermofisher). Search parameters included a no-enzyme setting, precursor (10 ppm) and product ion (0.05 Da) tolerances, a fixed modification of oxidation, N-terminal pyroglutamate, N-terminal acetylation, and C-terminal amidation. Data were filtered to include only protein identifications with a 1% false discovery rate (FDR) and at least one unique peptide. Peak area intensity of parental proteins in sorted cells was calculated in PEAKS. These mass spectrometry proteomics data have been deposited to the ProteomeXchange Consortium via the PRIDE partner repository with the dataset identifier PXD062039 and 10.6019/PXD062039.

### Secretion Assays

Differentiated organoids were removed from domes using ice-cold advanced DMEM/F-12 (ADF) and centrifuged at 400 g for 4 minutes. Organoids were washed twice for 15 minutes at 37°C with saline buffer supplemented with 1 mM glucose, 0.1% bovine serum albumin (BSA), 10 mM fluoxetine, and 10 mM 5-HT stabilizer (from kit). Approximately 30 organoids per well were distributed into V-bottom 96-well plates and incubated in duplicate or triplicate wells with test reagents (or vehicle control) dissolved in 100 mL of saline buffer for 1 hour at 37°C. Subsequently, plates were centrifuged at 2000 g for 5 minutes at 4°C, and supernatants collected into individual tubes were snap-frozen prior to analysis. Total 5-HT levels in the supernatants were measured by LDN Serotonin ELISA kit (LDN), which has a range of 0.015 to 2.5 ng/mL. Forskolin and IBXM (F + I, 10 mM and 100 mM, respectively) were used as positive control in all secretion experiments, and treatments were normalized to basal release in saline buffer without additions on the same plate. Average basal serotonin release across all experiments was 0.47 ± 0.3 ng/mL. Only compounds that activate receptors whose expression was detected by RNAseq and which were shown to induce either a calcium (Gq-coupled receptors or ion channels) or cAMP (Gs-coupled receptors) response and therefore were expected to induce serotonin release were tested (Table 1).

**Table 1:** Drugs Used for Secretions, Perfusions, Imaging, and Electrophysiological Studies

Reagent	Description	Source	Catalog no.
Adrenaline	Adrenoceptor agonist	Sigma-Aldrich	E4250
Amphotericin-B	Antifungal antibiotic (perforating agent)	Sigma-Aldrich	A2942
AR231453	GPR119 agonist	Sigma-Aldrich	SML1883
Clonidine hydrochloride	⍺2-adrenergic agonist	Tocris	0690/100
Cinnamaldehyde (CA)	TRPA1 agonist	Sigma-Aldrich	W228613
D-(+)-Glucose	Nutrient	Merck Life Science UK Limited	G7528-250G
Dimethyl sulfoxide (DMSO)	Solvent	Sigma-Aldrich	D8418
Exendin-4	GLP-1R agonist	Cambridge Bioscience	4027457.1000
Fluoxetine hydrochloride	SERT inhibitor	Fluorochem	F375072
Forskolin	Adenylate cyclase activator	Sigma-Aldrich	F6886
Fura2-AM	Cell permeable Ca^2+^ indicator	Invitrogen	F1221
GPBAR-A	G-protein bile acid receptor 1 agonist	Sigma-Aldrich	SML1207
Human glucose-dependent insulinotropic peptide (GIP)	GIPR agonist	Cambridge Bioscience	AS-61226-05
Human peptide YY (PYY) 3-36	NPY Y2 receptor agonist	Tocris	6288
Human cholecystokinin (CCK)	CCRAR agonist	Phoenix Pharmaceuticals	069-27
3-isobutyl-1-methylxanthine (IBMX ()	Phosphodiesterase inhibitor	Sigma-Aldrich	I7018
INSL5, short A- & B-chains	Rxfp4 agonist	Phoenix Pharmaceuticals	035-70A
Isovaleric acid	OR51E1 agonist	Acros Organics	156690025
Isopreterenol	Beta adrenergic agonist	Sigma-Aldrich	5984-95-2
L-Phenylalanine	Aromatic amino acid	Sigma-Aldrich	P5482-25G
L-Tryptophan	Aromatic amino acid	Sigma-Aldrich	T0254
Noradrenaline	Adrenoceptor agonist	Sigma-Aldrich	N5785
Pertussis toxin (PTX)	Catalyses ADP-ribosylation of the α subunits of Gi/o family	Sigma-Aldrich	70323-44-3
Sodium acetate trihydrate	Short-chain fatty acid	Sigma-Aldrich	6131-90-4
Sodium butyrate	Short-chain fatty acid	Sigma-Aldrich	B5887
Somatostatin 14 (SST)	SSTR1-5 agonist	Generon Ltd	AS-24277

### Calcium and cAMP Imaging

Live single-cell calcium imaging of Venus-expressing EC cells was performed as previously described after loading with Fura2-AM.^28^^,^^29^^,^^47^ cAMP-dependent FRET imaging was performed as described previously^23^ on TPH1-Epac1-S-H187 organoids. Briefly, differentiated organoids were removed from domes using ice-cold ADF and centrifuged at 400 g for 4 minutes. Pellets were treated with TryplE (Thermofisher) for 7 minutes at 37°C, following neutralization using heat inactivated fetal bovine serum (FBS) (Thermofisher) and centrifugation at 400 g for 4 minutes. Pellets were then resuspended in organoid media and plated in Matrigel-coated (4%) glass-bottom dishes (MatTek Corporation) 24 hours before experiments. For calcium imaging experiments, cells were loaded with Fura2-AM for 15 minutes at 37°C and 15 minutes at room temperature. Dishes were then washed 3 times with saline buffer and covered with foil to prevent bleaching. Cells were imaged using an inverted fluorescence microscope (Olympus IX71) with a 40× oil-immersion quartz-objective, coupled to an Orca-ER CCD camera (Hamamatsu). Cells were excited at 340/10 nm and 380/4 nm for 100 ms every 2 seconds using a Xenon arc lamp and a monochrmomator (Cairn Research controlled by MetaFluor software (Molecular Devices), and 340/380 ratios were calculated from background subtracted emissions recorded behind a 510-nm long pass emission filter. TPH1-Venus+ cells were identified (excitation at 480/10 nm) and 1 to 3 Fura2-positive cells were recorded per dish. For cAMP monitoring, 1 to 3 TPH1-Epac1-S-H187-positive cells/dish were recorded on a similar setup, consisting of an Olympus IX71 microscope and a xenon arc lamp/monochromator (Cairn), but in this case, emission was passed through an Optosplit II image splitter (Cairn Research) equipped with cyan fluorescent protein (CFP) and yellow fluorescent protein (YFP) emission filter sets, acquiring 470/24 nm and 535/30 nm respectively, before being recorded with an OrcaER CCD camera. Cells were excited at 435/10 nm, and CFP/YFP emission ratios were calculated after background subtraction using MetaFluor software. Depending on the known preferential coupling of their respective receptors, compounds were tested by either calcium imaging (Gq-coupled receptors) or cAMP imaging (Gs-coupled and Gi-coupled receptors) (Table 1).

### Electrophysiology

Patch clamp recordings of individual Venus-fluorescent cells were performed using 2D monolayer cultures 1 to 2 days after seeding, at room temperature in extracellular saline solution. Cells were seeded as described for imaging experiments, in plastic 35-mm bottom dishes (Corning Inc) pre-coated with 4% Matrigel. Patch pipettes were pulled from borosilicate glass (1.5 mm OD, 1.17 mm ID, Harvard Apparatus) to 3 to 7 MΩ using either a p-2000 puller (Sutter Instruments) or a PC-100 puller (Narishige, Japan) and fire polished using a microforge. Tips were coated with refined yellow beeswax for perforated patch clamp experiments.

Perforated patch current clamp recordings were performed using an Axopatch 200B amplifier connected through a Digidata 1440A A/D converter and visualized using pCLAMP software (Molecular Devices). Pipettes were filled with an intracellular solution consisting of (mmol/L): 76 K_2_S0_4_, 10 NaCl, 10 KCl, 10 HEPES, 55 sucrose, 1 MgCl_2_; adjusted to pH 7.2 with KOH, and the extracellular solution consisted of (mmol/L) 138 NaCl, 4.5 KCl, 10 HEPES, 4.2 NaHCO_3_, 2.6 CaCl_2_, 1.2 MgCl_2_, 1.2 Na_2_HPO_4_, 1 D-Glucose. A stock amphotericin-B solution dissolved in dimethyl sulfoxide (DMSO) was prepared fresh on the day of recording and diluted in intracellular solution to a final concentration of 200 μg/mL. Evoked action potentials were recorded in current-clamp mode by applying square current steps of increasing size (50 or 500ms, Δ2- 30 pA) from a baseline potential of −70mV.

Whole-cell voltage clamp capacitance recordings were performed using the Sine ± DC technique in the lockIn extension of a HEKA EPC-10 amplifier and Patchmaster software (HEKA).

For measurements of vesicular pool size, the intracellular solution consisted of (mmol/L): 123 glutamic acid, 40 Cs-HEPES, 2 Mg-ATP, 1 MgCl_2_, 17 NaCl, 0.26 EGTA, adjusted to pH 7.2 with CsOH and the extracellular solution consisted of (mmol/L) 145 NaCl, 2.8 KCl , 2 CaCl_2_, 1 MgCl_2_, 10 HEPES, 11 glucose. Osmolarity was adjusted to 300 to 310 mOsm. Pool size measurements were performed using a 10-pulse train protocol of 6 short 10-ms depolarizations followed by 4 longer 100-ms depolarizations (−70 to +20mV), with 300 ms interpulse interval. Capacitance was recorded using a 1 kHz sine wave with a peak amplitude of 35 mV superimposed at a holding potential of −70 mV before, between individual pulses, and for 9.5 seconds after the last depolarizing pulse. Analysis of capacitance recordings was performed using Igor Pro 8 (Wavemetrics), and capacitance changes were calculated between the average capacitance recorded over a 300-ms period before the first pulse and over a 300-ms period after the last 10 ms or 100 ms pulse or during the 9.5-seccond final recording time. The first 6 10-ms pulses should primarily activate the IRP, as only a very brief and limited influx of Ca^2+^ is induced during these pulses. The last 4 100-ms pulses allow a more sustained elevation of Ca^2+^ resulting in the release of the RRP, and eventually, over a longer time frame, a sustained release. Fusion of vesicles with the plasma membrane increases membrane surface area,^48^ which can be recorded as changes in the total membrane capacitance, with the capacitance change reflecting net vesicular fusion (exocytosis minus endocytosis). Of the 23 total human EC cells assessed, only 5 made it into the final analysis; 9 cells (39%) were excluded because they exhibited no exocytosis (defined by a lack of capacitance change despite the presence of robust inward currents), and 3 (13%) were excluded because they exhibited spontaneous exocytosis (defined by capacitance measurements that fluctuated without correlation to current traces). Of the remaining 11 cells that exhibited provoked exocytosis (defined by a clear capacitance increase following current peaks), 2 were excluded due to unstable patch measurements, and 4 were excluded because they required more than one application of the depolarization protocol before exocytosis was evident.

For capacitance measurements assessing potentiation of exocytosis, the intracellular solution consisted of (mmol/L) :125 caesium gluconate, 10 TEA-Cl, 10 NaCl, 1 MgCl_2_, 5 HEPES, 10 EGTA, 3 Mg-ATP, 0.5 GTP, adjusted to pH 7.2 with CsOH and the extracellular solution consisted of (mmol/L): 138 NaCl, 4.5 KCl , 10 HEPES, 4.2 NaHCO_3_, 2.6 CaCl_2_, 1.2 MgCl_2_, 1.2 Na_2_HPO_4_, 1 D-Glucose, supplemented with 200 μmol/l Isovalerate or 10 μmol/L of forskolin and 100 μmol/L of IBMX, dependent on recording condition. Capacitance recordings were performed using a 15-pulse train of square depolarizations (−60 to +0mV, 500 ms; with 300-ms interpulse capacitance measurements). Analysis of capacitance recordings was performed using RStudio (Posit), and capacitance changes were calculated between the average capacitance recorded over a 300-ms period before the first pulse and over a 300-ms period after the last pulse. All cells with stable conductance and access resistance demonstrating a change in capacitance after the pulse train were included in the analysis.

### Immunocytochemistry

Antibodies used were as follows; rabbit αGFP (1:1000, Nordic Biosite), goat α5-HT (1:1000, Immunostar), rabbit αCgA (1:50, Abcam), DαR555 (1:1000, ThermoFisher Scientific), and DαG633 (1:1000, ThermoFisher Scientific). Immunocytochemistry was performed on 2D monolayer cultures of human TPH1-venus organoids, which were prepared as described above. Forty-eight hours post-seeding, the cultures were washed 3 × 5 minutes in phosphate buffered saline (PBS) and fixed in 4% paraformaldehyde for 30 minutes. All washes and incubations were performed at room temperature in a rocker set to 12 rpm. Cultures were then washed 3 × 5 minutes in PBS and left in blocking solution (10% serum, 0.3% Triton X-100) for 45 minutes before being incubated in antibody solution containing primary antibodies (5% serum, 0.1% Triton X-100) for 2 hours. Cultures were then washed 3 times in PBS and incubated in antibody solution containing secondary antibodies for 1 hour. Cultures were washed a further 3 times in PBS and then incubated in 4',6-diamidino-2-phenylindole (DAPI) solution (0.1% DAPI in PBS) for 10 minutes, washed a further 3 times in PBS, and mounted using Aqua-Poly/Mount mounting media. Slides were imaged using a Zeiss Axio Observer microscope with an Axiocam 702 camera and a Colibri 5/7 LED light source within the ZEN Microscopy software (ZEISS), using consistent intensities and exposure times across samples.

### Buffers and Materials

Secretion assays and live fluorescent microscopy (Ca^2+^ and cAMP measurements) were carried out using saline buffer containing (in mmol/L): 138 NaCl, 4.5 KCl, 4.2 NaHCO_3_, 1.2 NaH_2_PO_4_, 2.6 CaCl_2_, 1.2 MgCl_2_, 10 HEPES; adjusted to pH 7.4 with NaOH. Glucose and test compounds were added as indicated. All chemicals were purchased from Sigma Aldrich if not stated otherwise.

### Statistical Analysis

Calcium (Fura2 ratios) and cAMP (FRET) data were not normally distributed, so expressed as median with 95% confidence interval (CI) shown in the figures and the IQRs stated in the text. Secretion data (5-HT accumulated in cell supernatant and changes in cell-capacitance) are presented as mean with 95% CI in the figures and mean ± SD stated in the text. Statistical tests were performed using GraphPad Prism v10, DESeq2 (RNA sequencing) or R v12. For calcium and cAMP imaging experiments, cells were included in the analysis if they showed responses to the positive control (KCl or forskolin/IBMX, respectively) of at least a 10% elevation. They were classified as ‘responders’ to specific test agents if the *z* score was >3 for at least 2 consecutive timepoints during perfusion of test substance; *z* score = [(*F*_*t*_ − mean *F*_b_)/SD *F*_b_], where *F*_*t*_ is the ratio (fura2 or FRET) at timepoint *t* during perfusion of the test reagent, *F*_*b*_ is the ratio under basal conditions, and mean *F*_*b*_ and SD *F*_*b*_ are calculated from 60 seconds of datapoints prior to test addition. For statistical determination of whether there was a significant calcium or cAMP response across all cells tested with a specific agent (including cells classified as nonresponders for this agent), Fura2 or FRET ratios in the presence of the test agent were normalized to the ratio under basal conditions measured for each cell. Statistically significant elevations above baseline were determined using a 1-sample Wilcoxon test (against a baseline of 1). Inhibitory effects of agents on cAMP in the presence of IBMX were assessed by Friedman test with post hoc Dunn’s multiple comparisons test. 5-HT secretion was normalized to the mean basal measurement in the same plate and significance tested by 1-sample *t*-test against a baseline of 1. Capacitance measurements under different conditions were compared by analysis of variance (ANOVA) with post hoc Dunnett’s multiple comparisons test.
